# The fast nucleation/growth of Co_3_O_4_ nanowires on cotton silk: the facile development of a potentiometric uric acid biosensor

**DOI:** 10.1039/d2ra03149c

**Published:** 2022-06-22

**Authors:** Munirah D. Albaqami, Shymaa S. Medany, Ayman Nafady, Mazhar Hussain Ibupoto, Magnus Willander, Aneela Tahira, Umair Aftab, Brigitte Vigolo, Zafar Hussain Ibupoto

**Affiliations:** Department of Chemistry, College of Science, King Saud University P. O. Box 2455 Riyadh 11451 Saudi Arabia anafady@ksu.edu.sa; Department of Chemistry, Faculty of Science, Cairo University Cairo Egypt; Department of Zoology, Shah Abdul Latif University Khairpur Mirs Pakistan; Department of Science and Technology, Campus Norrköping, Linköping University SE-60174 Norrköping Sweden; Dr. M.A Kazi Institute of Chemistry, University of Sindh Jamshoro 76080 Sindh Pakistan zaffar.ibhupoto@usindh.edu.pk; Department of Metallurgy and Materials Engineering, Mehran University of Engineering and Technology 76080 Jamshoro Sindh Pakistan; Université de Lorraine, CNRS, IJL F-54000 Nancy France

## Abstract

In this study, we have used cotton silk as a source of abundant hydroxyl groups for the fast nucleation/growth of cobalt oxide (Co_3_O_4_) nanowires *via* a hydrothermal method. The crystal planes of the Co_3_O_4_ nanowires well matched the cubic phase. The as-synthesized Co_3_O_4_ nanowires mainly contained cobalt and oxygen elements and were found to be highly sensitive towards uric acid in 0.01 M phosphate buffer solution at pH 7.4. Importantly, the Co_3_O_4_ nanowires exhibited a large surface area, which was heavily utilized during the immobilization of the enzyme uricase *via* a physical adsorption method. The potentiometric response of the uricase-immobilizing Co_3_O_4_ nanowires was measured in the presence of uric acid (UA) against a silver/silver chloride (Ag/AgCl) reference electrode. The newly fabricated uric acid biosensor possessed a low limit of detection of 1.0 ± 0.2 nM with a wide linear range of 5 nM to 10 mM and sensitivity of 30.6 mV dec^−1^. Additionally, several related parameters of the developed uric acid biosensor were investigated, such as the repeatability, reproducibility, storage stability, selectivity, and dynamic response time, and these were found to be satisfactory. The good performance of the Co_3_O_4_ nanowires was verified based on the fast charge-transfer kinetics, as confirmed *via* electrochemical impedance spectroscopy. The successful practical use of the uric acid biosensor was demonstrated based on the recovery method. The observed performance of the uricase-immobilizing Co_3_O_4_ nanowires revealed that they could be considered as a promising and alternative tool for the detection of uric acid under both *in vitro* and *in vivo* conditions. Also, the use of cotton silk as a source of abundant hydroxyl groups may be considered for the remarkably fast nucleation/growth of other metal-oxide nanostructures, thereby facilitating the fabrication of functional electrochemical devices, such as batteries, water-splitting devices, and supercapacitors.

## Abstract 2

In this study, we have used cotton silk as a source of abundant hydroxyl groups for the fast nucleation/growth of cobalt oxide (Co_3_O_4_) nanowires *via* a hydrothermal method.

## Graphical abstract

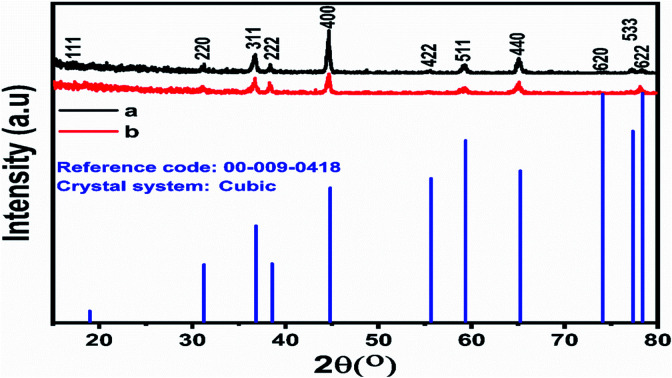

## 1. Introduction

The potentiometric method is a versatile electroanalytical approach for quantifying the amounts of small ions like H^+^, K^+^, and F^−^ in solution.^1^ The potentiometric method does not involve current bias like other electrochemical methods, including chronoamperometry, differential pulse voltammetry, and cyclic voltammetry. This highlights the superiority of the potentiometric method and enables it to be used in biological matrices without causing damage. The potentiometric technique has a very swift response, is very simple, and does not require skilled operators; therefore, it can be used for the monitoring of uric acid *in situ*.^2–6^ Several types of potentiometric analysis are carried out annually *via* the use of classical membrane-based ion-selective electrodes. Today, in the fields of medical diagnosis and disease treatment, there is an immediate need to design and develop highly functional devices for the identification, detection, and quantification of small biomolecules, which play important roles in our body. The proposed devices should be fast-responding, facile, sensitive, selective, and highly accurate during the diagnosis of various diseases.

Uric acid (UA) is an antioxidant and present in urine and serum. UA is excreted by the kidneys; it is an end product resulting from the metabolism of purine nucleotides and their derivatives, and it causes many biological changes in the body.^7,8^ A high concentration of UA in bodily fluids^9^ is linked to many diseases, including gout,^10^ hyperuricemia, Lesch–Nyhan syndrome,^11^ and kidney and cardiovascular complications.^7^ These medical issues confirm that the regular monitoring of UA is very essential and, therefore, various analytical methods are used to determine UA levels, such as high-performance liquid chromatography (HPLC),^12^ spectrophotometry,^13^ and chemiluminescence- and enzyme-based biosensors.^14,15^ UA biosensors have begun to receive more attention due to their numerous advantages, such as simplicity, cost effectiveness, high sensitivity, and excellent selectivity.^16–18^ Uricase oxidase accelerates the oxidation of UA and transforms it to allantoin. The high sensitivity and selectivity of uricase oxidase allow it to act as an excellent indicator for the development of efficient UA biosensors.^19^ One strategy involves transforming the biochemical reaction to an electrical signal using enzyme-based amperometric biosensors for the quantification of redox species. A second strategy involves using electron-transfer mediators between the active sites of enzymes and the electrode.^20,21^ However, enzyme-based approaches have shown limited effectiveness, slow electrode kinetics, and high overpotentials, ultimately impairing charge transfer and suffering from the issue of interference from other easily oxidized substances. Also, the leaching of soluble mediators from the electrode surface is unavoidable.

These major challenges relating to the development of UA biosensors have been identified.^22^ The use of an efficient transducer is a pre-requirement for the development of UA biosensors, and it can easily overcome these limitations. Therefore, the synthesis of new functional transducers based on nanostructured materials is appreciated by the scientific community in the fields of materials and medical science. The use of nanostructured transducers enables the production of strong electrical signal due to fast electron communication and high surface areas; therefore, they can act as excellent host materials for the loading of enzymes. The excellent functionality of nanostructured materials is related to their high surface-to-volume ratios and rapid electron-transfer activities.^23^ The high performance of nanostructured materials is also governed by the size, chemical composition, specific surface area, catalytic activity, and morphology.^24^

A wide range of nanostructured materials, like noble metals and metal oxides, has been prepared and used for sensing applications as transducers, a main component of sensing devices.^25–39^ Cobalt oxide (Co_3_O_4_) has many advantages, including high biocompatibility, a narrow band gap, excellent stability, and low cost, and it can be produced from earth-abundant materials. These features of Co_3_O_4_ have been widely studied for electrochemical applications, like its use in sensors, supercapacitors, and batteries.^30,41–46^ It has also been well studied for biomedical applications.^40^ Also, Co_3_O_4_ exhibits significant stability and selectivity, which are desirable and have been intensively studied for sensing applications.^47^ For improving the performance of Co_3_O_4_, various composite systems have been developed and used for a wide range of sensing applications.^48–58^ Most of these composite systems involve multistep synthesis and possess complex structures. This is the reason why new and simple strategies are required for the facile synthesis of Co_3_O_4_ nanostructures with superior or equal performance to these previously reported composites based on Co_3_O_4_. Still, the performance of Co_3_O_4_ is limited for electrochemical applications due to its poor electrical conductivity, lack of catalytic sites, and tendency to undergo self-aggregation in aqueous solution. Therefore, more attention is needed to synthesize Co_3_O_4_ nanostructures with improved electrical conductivity, a high density of catalytic sites, and dispersibility in aqueous solution. For this purpose, we used a naturally abundant source of hydroxyl groups, cotton silk, which swiftly accelerated the nucleation rate of well-oriented Co_3_O_4_ nanowires. The calcination of cotton silk provides an abundant source of carbon in the chemical environment of the Co_3_O_4_ nanostructures, which can improve the dispersion of Co_3_O_4_ in aqueous solution. This can further enhance the electrochemical performance of Co_3_O_4_ nanostructures. Conclusively, cotton silk improves Co_3_O_4_ from two aspects: helping to control the morphology and the dispersion. Therefore, the present study has overcome the main limitations of Co_3_O_4_ during electrochemical processes and increased the performance of nanostructured Co_3_O_4_ for the development of a potentiometric uric acid biosensor.

In this study, we obtained a high surface area and abundant hydroxyl groups for the fast nucleation of Co_3_O_4_ nanowires using a two-step approach. The Co_3_O_4_ nanostructures were characterized *via* scanning electron microscopy (SEM), energy dispersive spectroscopy (EDS), and X-ray diffraction (XRD), and they were functionalized with the enzyme uricase oxidase through electrostatic attraction. Then, the uricase-immobilizing nanostructured Co_3_O_4_ was used for the potentiometric determination of UA in phosphate buffer solution at pH 7.4. A performance evaluation of the UA biosensor was carried out in terms of stability, selectivity, linear range, reproducibility, and response time. The obtained results confirm that the proposed material can be used as an alternative transducer for the development of practical sensing devices.

## 2. Experimental details

### 2.1. Chemical reagents

The enzyme uricase oxidase (E.C. 1.7.3.3) with activity of 25 units/1.5 mg was obtained from *Arthrobacter globiformis*; β-d-glucose (99.5%), uric acid (99.8%), 25% glutaraldehyde, ascorbic acid, dopamine, urea, ethanol, cobalt chloride hexahydrate, potassium chloride, sodium chloride, sodium biphosphate (Na_2_HPO_4_), sodium hydroxide, hydrochloric acid, and potassium dihydrogen phosphate (KH_2_PO_4_) were purchased from Sigma-Aldrich, Karachi, Pakistan. All the chemicals were of analytical grade and used without further purification. 0.01 M phosphate buffer solution at pH 7.4 was prepared *via* mixing 0.01 M sodium chloride, 0.02 M potassium chloride, 0.001 M KH_2_PO_4_, and 0.005 M Na_2_HPO_4_ in deionized water. Deionized water was used as the main solvent medium. Cotton silk was obtained from a local market in Hyderabad, Sindh, Pakistan.

### 2.2. Preparation of Co_3_O_4_ nanowires on cotton silk *via* a hydrothermal process

**Scheme 1:**
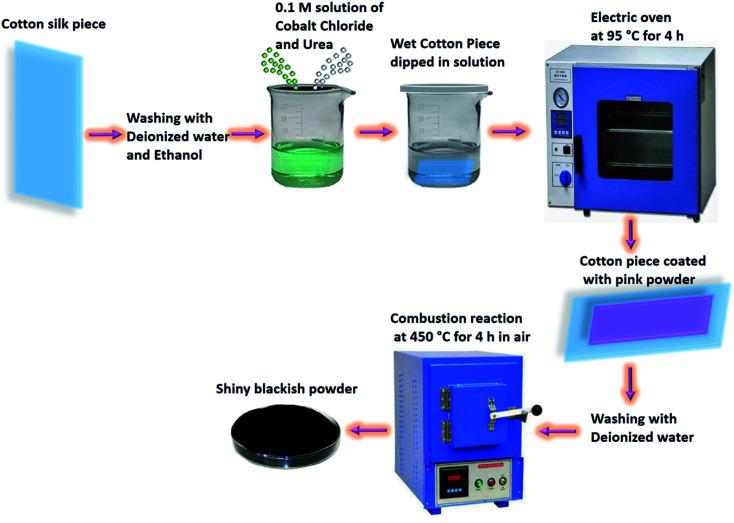
The synthesis process for the prepared Co_3_O_4_ nanostructures.

The fabrication of Co_3_O_4_ nanostructures on cotton silk was done through a hydrothermal process followed by a combustion reaction (Scheme 1). Prior to the hydrothermal process, the cotton silk was washed gently with deionized water and ethanol. Then, a wet piece of cotton silk was dipped into a 0.1 M solution of cobalt chloride hexahydrate and urea until it settled at the bottom of the beaker. Afterwards, the cobalt precursors containing the wet piece of cotton silk were covered tightly with an aluminum sheet and put into a preheated electric oven at 95 °C for 4 h. Then, a pink layer coated the cotton silk, which was washed several times with deionized water and left to dry overnight. Then, a combustion reaction was carried out on the pink product deposited on the cotton silk for 4 h at 450 °C in air. A shiny blackish product was successfully prepared. The synthesis of pristine Co_3_O_4_ nanostructures was carried out *via* the same method but without the use of cotton silk.

The morphology and composition of the as-obtained Co_3_O_4_ nanostructures were studied through the use of low-resolution scanning electron microscopy operated at 3 kV. Elemental analysis of the Co_3_O_4_ nanostructures was done using energy-dispersive spectroscopy equipped with SEM. Powder X-ray diffraction was used to identify the crystal planes and purity of the as-prepared Co_3_O_4_ nanostructures. The XRD measurements were done using X-rays from CuKα radiation (*λ* = 1.54050 Å) at 45 mA and 45 kV. Potentiometric experiments were performed using a pH meter (METTLER TOLEDO) and the dynamic response was measured through a potentiostat supplied from the Netherlands. Charge-transfer evaluation was carried out *via* electrochemical impedance spectroscopy (EIS) from 50 kHz to 10 Hz, at a sinusoidal potential of 5 mV, with zero supplied potential, at room temperature, in 0.1 mM uric acid prepared in 0.01 M phosphate buffer solution at pH 7.4. Z-view software was used to fit the experimental data to an equivalent circuit to check the reliability of the measured data.

### 2.3. Modification of uricase-immobilizing Co_3_O_4_ nanostructures onto a glassy carbon electrode

The cleaning of the glassy carbon electrode was done according to our previously published work.^59^ Briefly, the cleaning of the glassy carbon electrode was done with 5 μm alumina paste and silica paper, and then it was washed several times with deionized water and dried under blowing air.

The potentiometric responses of pristine and cotton-silk-deposited Co_3_O_4_ nanostructures were measured in 0.01 M phosphate buffer solution at pH 7.4. The immobilization of uricase onto 10 mg of Co_3_O_4_ nanostructures was done in phosphate buffer solution containing 10 mg of uricase and 100 μL of 1% glutaraldehyde. The soaking of Co_3_O_4_ nanostructures in uricase enzyme solution was performed for 30 min in order to fully cover the Co_3_O_4_ nanostructures with enzyme molecules. Then, 10 μL of uricase-enzyme-immobilizing Co_3_O_4_ nanostructures with a loading mass of 0.2 mg was deposited on the cleaned glassy carbon electrode *via* a drop-casting process. Then, the modified electrode was dried at room temperature followed by overnight soaking in phosphate buffer solution at pH 7.4 to get a stable potential output response. Then, the soaked electrode was dried under atmospheric conditions and was ready to carry out the sensing of uric acid oxidation. A glassy carbon electrode with an area of 3 mm was used, and the thickness of the sensing layer was around 50 nm.

The modified electrode was labelled as the working electrode and its output potential was measured against a reference silver/silver chloride (Ag/AgCl) electrode filled with 3 M KCl. A two-electrode set-up was used in an electrochemical cell containing a specific concentration of uric acid prepared in 0.01 M phosphate buffer solution at pH 7.4. For the stock solution, we first heated uric acid (UA) in 1 M NaOH aqueous solution at 75 °C for 3 h, and then we made stock solution with a concentration of 15 mM in phosphate buffer solution, adjusting the pH to around 7.4 using concentrated hydrochloric acid solution. Afterwards, we used a dilution equation for the preparation of low concentrations of UA. All the studies were done at room temperature, other than those investigating the effects of temperature on the potentiometric signal.

## 3. Results and discussion

### 3.1. The crystallinity, morphology, and composition of the Co_3_O_4_ nanostructures

**Fig. 1:**
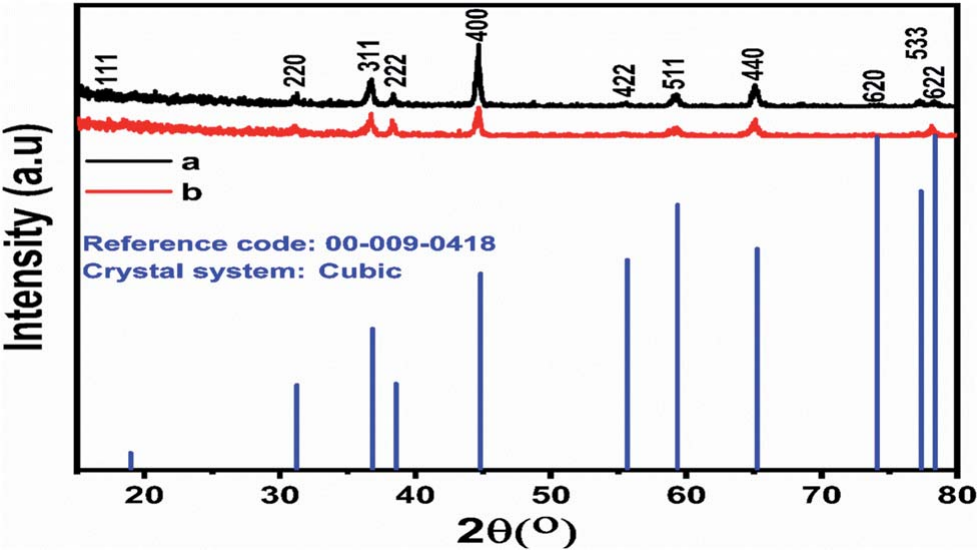
Powder XRD patterns of (a) pristine Co_3_O_4_ platelets and (b) Co_3_O_4_ nanowires obtained with cotton silk.

The typical diffraction patterns of pristine and cotton-silk-deposited Co_3_O_4_ nanostructures are shown in Fig. 1a and b. The crystal planes of the pristine Co_3_O_4_ nanostructures indicate a pure cubic phase, and this is fully supported by the reference card no. 00-009-0418. The cotton-silk-deposited Co_3_O_4_ nanostructures show a similar diffraction pattern but the peaks are slightly shifted towards higher angles, as shown in Fig. 1b. The crystal planes of the cotton-silk-deposited Co_3_O_4_ nanostructures are also in good agreement with the above reference card. The XRD study has verified that no carbon-based impurities from cotton were found, suggesting that cotton can be used as a high-surface-area substrate to deposit large-surface-area metal-oxide nanostructures.

**Fig. 2:**
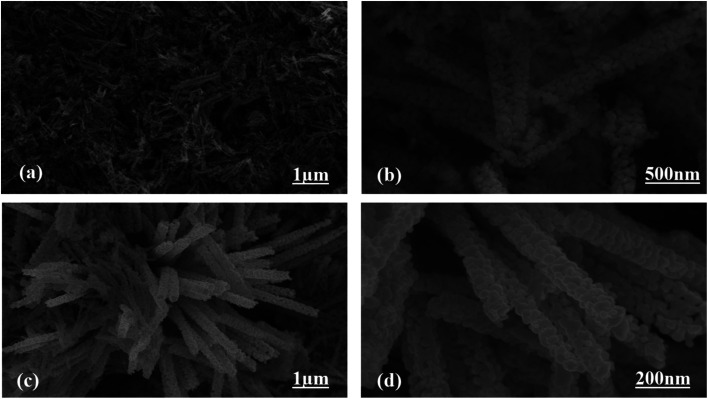
Typical SEM images at different magnifications: (a and b) pristine Co_3_O_4_ platelets and (c and d) Co_3_O_4_ nanowires obtained with cotton silk.

The morphologies of the as-prepared Co_3_O_4_ nanostructures were investigated *via* SEM analysis, as shown in Fig. 2. The pristine Co_3_O_4_ nanostructures possess platelet-like morphology, consisting of self-assembled nanoparticles, as shown in Fig. 2a and b. The length of the Co_3_O_4_ platelets is on the micron-scale, and the nanoparticles have a size of 100 nm. The use of cotton silk as a high-surface-area substrate has led to the evolution of nanowire-like Co_3_O_4_ nanostructures. Also, these Co_3_O_4_ nanowires are a few microns in length with an average diameter of 100–200 nm. The nanowires evolved from the self-assembly of nanoparticles. The evolution of nanowires might be due to the existence of plenty of hydroxyl groups in the cotton silk, which accelerated the growth kinetics through swift nucleation. Furthermore, the presence of hydroxyl groups on the substrate surface could also be useful for binding the metal ions, which act as nucleation units for the development of metal-hydroxide crystals, thereby not only enhancing the growth kinetics but also determining the evolution of the morphology.

**Fig. 3:**
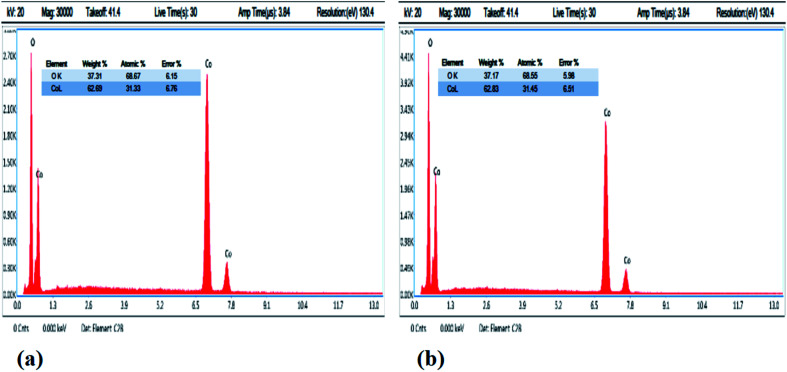
EDS spectra: (a) pristine Co_3_O_4_ platelets and (b) Co_3_O_4_ nanowires.

The composition of the as-prepared Co_3_O_4_ nanostructures was also analyzed using EDS analysis, as shown in Fig. 3a and b. The pristine Co_3_O_4_ nanostructures and cotton-silk-deposited Co_3_O_4_ nanowires showed only cobalt and oxygen as the main elements, as shown in Fig. 3a and b. The point of interest is that the relative amount of oxygen is lower in the Co_3_O_4_ nanowires, indicating the presence of more oxygen vacancies in the sample, as shown in Fig. 3b.

### 3.2. The potentiometric response of uricase-immobilizing Co_3_O_4_ nanostructures towards the sensing of uric acid

The set-up of the standard two-electrode cell can be described as follows:Co_3_O_4_|Co^3+^/Co^2+^ PBS sol.‖Ag/AgCl PBS sol.|Ag

**Fig. 4:**
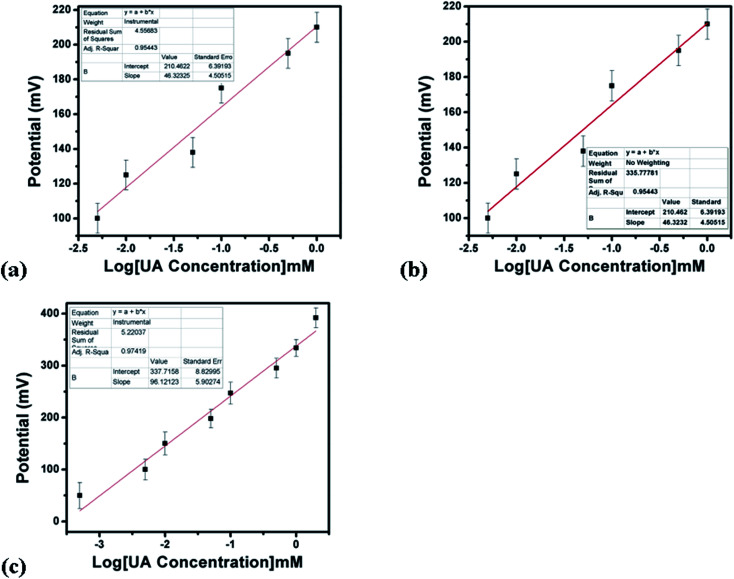
The potentiometric responses of (a) uricase-immobilizing pristine Co_3_O_4_ platelets, (b) Co_3_O_4_ nanowires without uricase enzyme immobilization, and (c) uricase-immobilizing Co_3_O_4_ nanowires to various concentrations of uric acid in 0.01 M phosphate buffer solution at pH 7.4.

The output potential of the electrochemical cell depends on the ionic charge present in the electrolytic solution, and the output potential response could be related to the concentration of charged species in the test solution based on calibration fitting. The Co_3_O_4_ nanostructures were used to immobilize uricase enzyme, then they were used for the detection of UA. It has been well established that uricase specifically oxidizes uric acid; consequently, the generation of different charged species is always expected. The role of the Co_3_O_4_ nanowires in this configuration is not only to provide a high surface-to-volume ratio for the heavy loading of uricase, as its role as a co-catalyst and promoter of uricase is obvious due to the strong potential output signal during the oxidation of UA. Potentiometric studies were initiated with a soaked modified electrode in 0.01 M phosphate buffer solution at pH 7.4 and a stable output potential was noticed; the addition of 1 nM uric acid resulted in an unstable signal, but the further addition of 5 nM uric acid resulted in a stable and repeatable signal. Therefore, a calibration curve was plotted from 5 nM to 10 mM, as shown in Fig. 4c. Fig. 4a shows the potentiometric response of uricase-immobilizing pristine Co_3_O_4_ platelets, indicating a poor output signal and poor analytical performance. The narrow linear range of 0.005–1 mM for uric acid and the regression coefficient of 0.95 cast doubts on the accuracy and precision of a uric acid biosensor based on uricase-immobilizing pristine Co_3_O_4_ nanostructures. The poor performance of pristine Co_3_O_4_ nanostructures is associated with the poor and weak immobilization of uricase due to the randomly oriented morphology of the Co_3_O_4_ nanostructures. Co_3_O_4_ nanowires without immobilization were also tested for the detection of UA, as shown in Fig. 4b. It is obvious that the Co_3_O_4_ nanowires have a relatively increased value of output potential compared with the pristine Co_3_O_4_ platelets, indicating that a well-oriented morphology has better compatibility for interactions with uric acid molecules and contributes towards fast electron communication. However, the performance of Co_3_O_4_ nanowires without the loading of uricase is still weak; therefore, we aimed to use uricase immobilized on the surface of Co_3_O_4_ nanowires *via* physical adsorption for the development of a sensitive and selective potentiometric uric acid biosensor. The activity of the Co_3_O_4_ nanowires is superior in this case due to the high surface-to-volume ratio, allowing the excellent immobilization of uricase, as shown in Fig. 4c. The potentiometric response of the proposed Co_3_O_4_ nanowires is highly sensitive to the analyzed substrate compound and throughout the study, the modified electrode follows the Nernst equation:*E* = *E*^0^ − 0.05916 V/*n* log[reduced]/[oxidized]

The generation of the output potential on the surface of uricase-immobilizing Co_3_O_4_ nanowires could be attributed to the co-catalytic and promoter role of the Co_3_O_4_ nanowires towards the oxidation of uric acid.

**Scheme 2:**
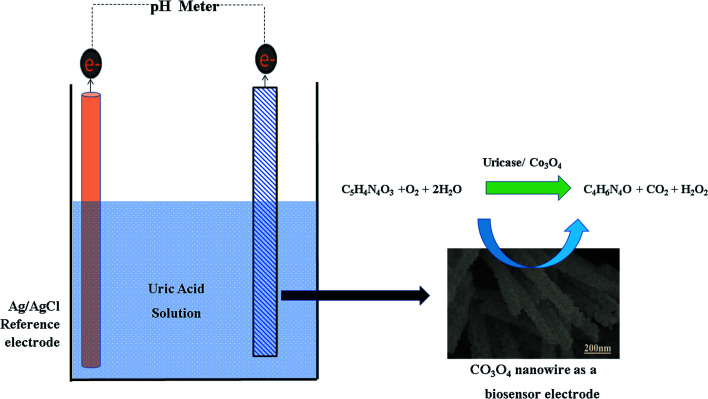
The sensing mechanism of UA with uricase immobilized onto Co_3_O_4_ nanostructures.

For the measurement of the uric acid concentration using the proposed uric acid biosensor, the biosensor electrode should be associated with an excellent range.^60^ The output potential of the modified electrode was evaluated at various uric acid concentrations ranging from 5 nM to 10 mM prepared in phosphate buffer solution at pH 7.4, as shown in Fig. 4c. The modified electrode demonstrated excellent analytical features, such as a regression coefficient of 0.99, a wide linear range, and a low limit of detection of 1.0 ± 2 nM. The potentiometric curve exhibited excellent correlation with log(uric acid concentration), as shown in Fig. 4c. The limit of detection and limit of quantification of 1.0 ± 0.2 nM were calculated according to a previously published work.^61^ The modified electrode showed a quick response time and a sensitivity of 30.5 mV dec^−1^. From Fig. 4c, the Nernst factor was observed to be about 30.5 mV per decade, as uric acid is a divalent molecule, as shown in Scheme 2. The Nernst factor estimated from the plot is (61/2) mV per decade, which equals 30.5 mV per decade (±1–3 mV).

The linear range, response time, and sensitivity of uricase-immobilizing Co_3_O_4_ nanowires are superior compared with pristine uricase-immobilizing Co_3_O_4_ platelets due to the well-oriented morphology, high surface-to-volume ratio, and favorable and highly compatible surface for the high-level loading of uricase enzyme molecules. It has been found that one-dimensional nanowires are associated with fast charge transport, and it is widely accepted that Co_3_O_4_ nanowires with well-controlled morphology are needed for the development of electrochemical devices. Moreover, Co_3_O_4_ offers unique p-type character for frequent interactions with the three oxygen atoms of uric acid, which ultimately may improve the reaction kinetics on the surface of the Co_3_O_4_ nanowires; therefore, we observed outstanding potentiometric performance. Furthermore, the sign of the output potential during the potentiometric determination of uric acid can be attributed to equilibrium established between the electrode and the analyte. UA is an easily oxidizable compound, therefore it might oxidize differently on various electrodes. Also, uric acid is an acid. This means that it can work as a dopant, *etc.* Hence, we should not be surprised that the sign of the output potential can be either positive or negative.

**Fig. 5:**
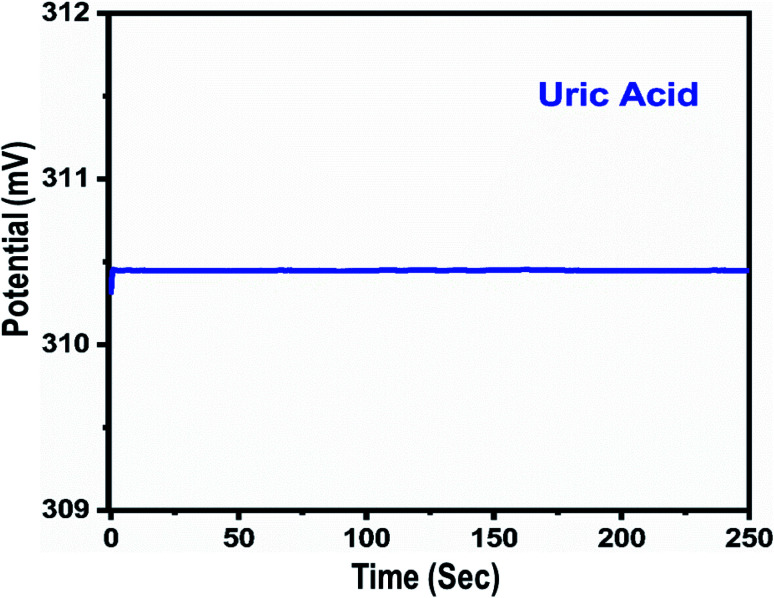
The dynamic potentiometric response of uricase-immobilizing Co_3_O_4_ nanowires to 1 mM uric acid in 0.01 M phosphate buffer solution at pH 7.4.

**Fig. 6:**
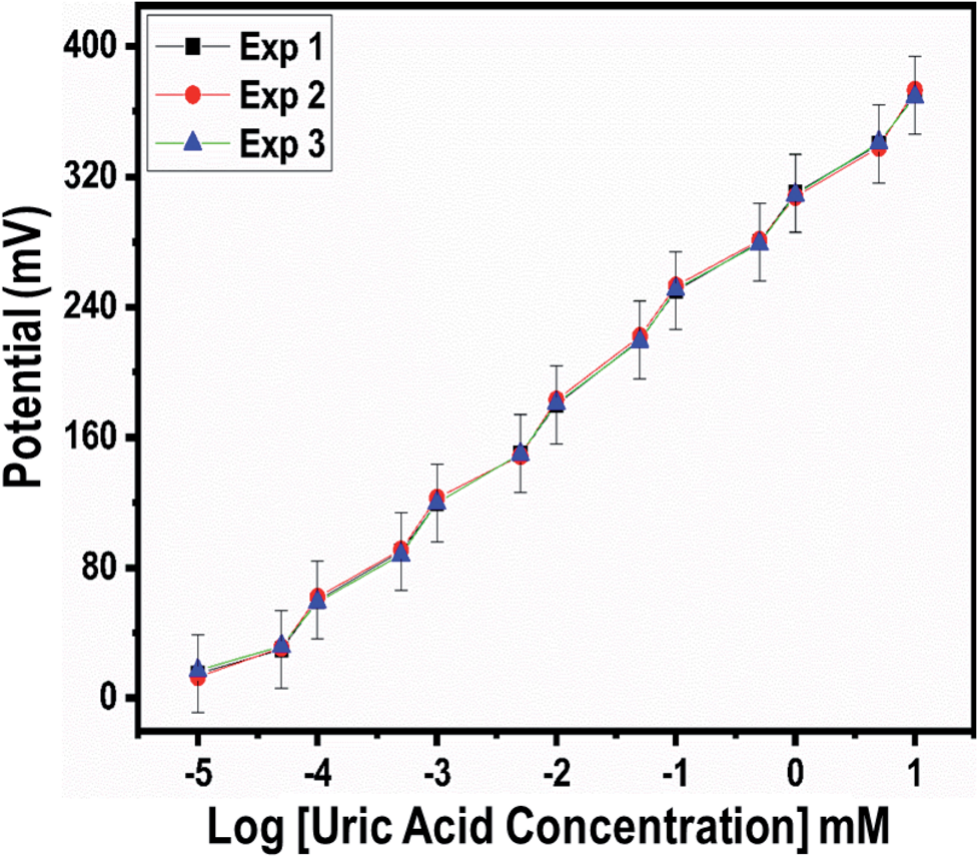
Repeatability experiments for uricase-immobilizing Co_3_O_4_ nanowires in 0.01 M phosphate buffer solution at pH 7.4.

A fast and sensitive electroanalytical method is always needed to monitor bioactive compounds from biological samples to ensure rapid biomedical diagnosis and treatment. For this purpose, the response time of the modified electrode was monitored in 1 mM uric acid, as shown in Fig. 5. The response time describes the kinetics and sensitivity of the modified electrode towards the sensing of a specific concentration of uric acid, and the proposed uric acid biosensor based on uricase-immobilizing Co_3_O_4_ nanowires exhibited a fast response time of less than 1 s. Additionally, the response time study also shows that the modified electrode is stable and the output potential did not change with time, as shown in Fig. 5. The repeated use of an electrode is another way to confirm the stability of a sensing layer on a glassy carbon electrode and to determine the number of times the modified electrode can be used. The use of the same modified electrode over several cycles is worth investigating and, therefore, we studied the repeatability of the modified electrode three times on alternative days using the same dynamic uric acid concentration range. We found that the presented electrode shows highly repeatability and there were no significant changes in sensitivity, linear range, and limit of detection, as shown in Fig. 6, confirming the high stability and excellent repeatability of the response from the sensing layer on the glassy carbon electrode. The repeatability experiments have verified the excellent performance of the modified electrode due to the firm adhesion and high biocompatibility between uricase and the surface of well-oriented Co_3_O_4_ nanowires. The inter-electrode response of a modified electrode is very essential to study, as it can confirm the accuracy and precision of the modification process. The quantitative responses of different modified electrodes prepared under the same conditions through an inter-electrode study can be used to determine figures of merit for a modified electrode in terms of the reliability of its analytical performance.

**Fig. 7:**
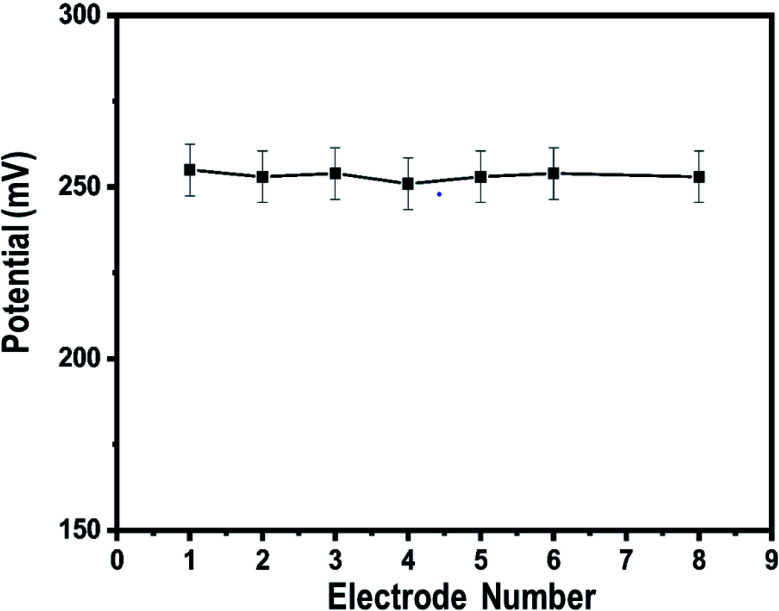
Inter-electrode responses of uricase-immobilizing Co_3_O_4_ nanowires to 0.1 mM uric acid in 0.01 M phosphate buffer solution at pH 7.4.

Therefore, we studied the similarity of the responses of seven independently produced Co_3_O_4_ nanowire electrodes in 0.1 mM uric acid testing solution, as shown in Fig. 7.

**Fig. 8:**
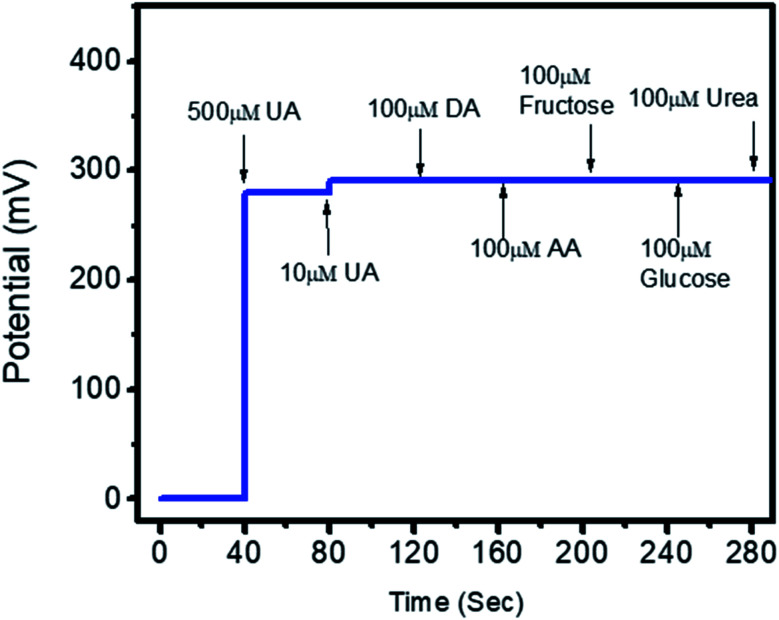
The selectivity response of uricase-immobilizing Co_3_O_4_ nanowires in the presence of 0.5 mM and 0.01 mM uric acid and various common interfering substances (0.1 mM urea, fructose, ascorbic acid, dopamine, and glucose) in 0.01 M phosphate buffer solution at pH 7.4.

The potentiometric responses indicate that the responses of the different electrodes are very similar, with standard deviation of less than 5%, revealing excellent analytic performance. Thus, the Co_3_O_4_ nanowires could be considered as a potential and alternative material for immobilizing uricase on a large scale for the practical monitoring of uric acid. Selectivity is one of the main parameters used to evaluate the performance of a uric acid biosensor, as some interfering substances, such as ascorbic acid, dopamine, and glucose, can influence the performance of a biosensor. Therefore, we studied the effects of 0.1 mM concentrations of these substances on the output potential of the uric acid biosensor in the presence of 0.01 mM uric acid using a sequential addition method, as shown in Fig. 8.

**Fig. 9:**
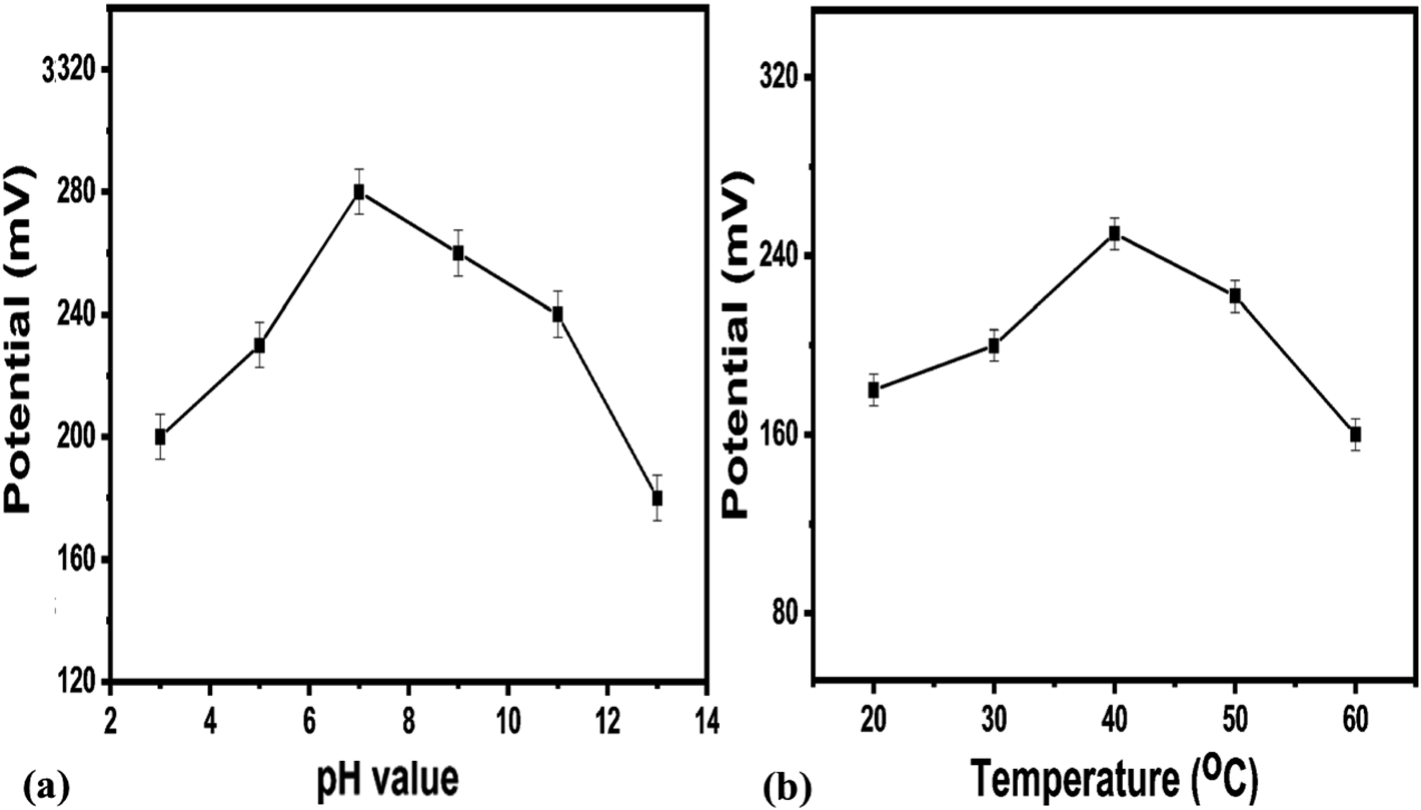
(a) The effects of testing solution pH on the potentiometric response of uricase-immobilizing Co_3_O_4_ nanowires in 0.5 mM uric acid in 0.01 M phosphate buffer solution. (b) The effects of temperature on the potentiometric response of uricase-immobilizing Co_3_O_4_ nanowires in 0.1 mM uric acid in 0.01 M phosphate buffer solution at pH 7.4.

It is clear from this study that Co_3_O_4_ nanowires functionalized with uricase have high selectivity towards uric acid sensing; thus, the modified electrode may be of great interest under *in vivo* conditions. This study has confirmed that negligible interference was observed during uric acid detection, and no significant changes in the output potential were noticed when interfering substances were added to the uric acid solution. Despite the generation of charge after the addition of these interfering substances, this might be counterbalanced by the ionic strength of the phosphate buffer solution; thus, this charge has a negligible impact on producing output potential, as shown in Fig. 8. The outstanding selectivity of the presented uric acid biosensor could be further attributed to the selectivity of uricase for uric acid and lower compatibility between Co_3_O_4_ nanowires and the interfering substances. The pH of the analyzed solution can have an influence on the activity of the sensing layer of the electrode in terms of the Co_3_O_4_ material stability, decreasing the effectiveness of uricase at certain pH values. As the response of the uricase-modified electrode can be affected upon changing the pH of the electrolyte, we studied the effects of pH on the biosensor signal in 0.5 mM uric acid, as shown in Fig. 9a. This study showed an optimum pH of around 7.0 for the modified electrode where it showed the maximum output potential. Also, it is reported that the activity of uricase is relatively high in the pH range of 8.5 to 9.2.^60^ We have already reported a pH sensor based on Co_3_O_4_.^62^ We studied the effects of pH on the EMF of pristine Co_3_O_4_ nanowires for pH sensor purposes, however, the reported work suggested that pH has significant effects on the stability of Co_3_O_4_;^60^ therefore, for this report, we studied the effects of pH on the performance of the uric acid biosensor based on Co_3_O_4_ nanowires. It was observed to be unstable under highly alkaline and acidic conditions due to dissolution and etching effects, as shown in Fig. 9a. Also, there is a possibility under highly alkaline conditions that interactions between hydroxyl groups and uricase might alter the kinetics of UA oxidation, consequently lowering the performance of the proposed biosensor. Keeping in mind these limitations of the sensing layer and the physiological conditions during uric acid detection in real samples, we studied the biosensing evaluation parameters of our proposed Co_3_O_4_-nanowire-based uric acid biosensor at a pH value of around 7.4.^53,58^

A pH level of 7.4 is close to that of the pH of human body fluids; therefore, all the studies were done at this pH except for the pH study. For biosensors based on bio-sensitive membranes, the influence of environmental temperature is a big challenge due to the denaturation of bio-sensitive membranes due to the stability of antibodies, enzymes, nucleases, *etc.* Thus, to confirm the storage conditions and to enhance the lifetime of the biosensor, we evaluated the performance of the uric acid biosensor at various temperatures, as shown in Fig. 9b. The results suggest that the optimum temperature for the presented uric acid biosensor is around 40 °C, with the biosensor having the maximum potential output at this temperature. Additionally, at higher temperatures, the activity of the sensing layer was decreased to the denaturation of uricase. Therefore, we measured and observed the potentiometric response at 20 °C to prevent the denaturation of uricase and to avoid the evaporation of the testing solution. An acceptable lifetime needs to be shown by the proposed uric acid biosensor during performance evaluation. The storage period of uricase-immobilizing nanowires obtained using cotton silk was also studied *via* monitoring the linear range, limit of detection, and sensitivity, as given in Table 1. It is obvious that the proposed modified electrode can be used for a time of more than 6 weeks if suitable storage conditions are maintained. This study suggests that the enzyme activity remains almost the same even after 6 weeks due to the possible role of the highly biocompatibility Co_3_O_4_ nanowires. We stored the modified electrode at 4 °C in a refrigerator when not in use.

**Table 1:** Performance of uricase-immobilizing Co_3_O_4_ nanowires after storage for different lengths of time

No of weeks	Linear range (mM)	Slope (mV dec^−1^)	Limit of detection (mM)
1	0.0005–10	61.05	0.0001
2	0.0007–10	61.02	0.0003
3	0.0002–10	60.5	0.0004
4	0.0006–9.5	60.8	0.0003
5	0.0003–10	61	0.0001
6	0.0005–10	60.7	0.0002

The activity of uricase-immobilizing Co_3_O_4_ nanowires was also compared with recently reported non-enzymatic uric acid sensors based either on Co_3_O_4_ or other materials. It is obvious that the presented approach has superior performance than many of the published results due to its wide linear range, limit of detection, and, most importantly, simple and easy approach for the synthesis of the nanostructured materials. The low cost and environmentally friendly synthesis of the Co_3_O_4_ nanowires also strengthen the case for the scalable fabrication of this potentiometric uric acid biosensor.^63–65^

**Fig. 10:**
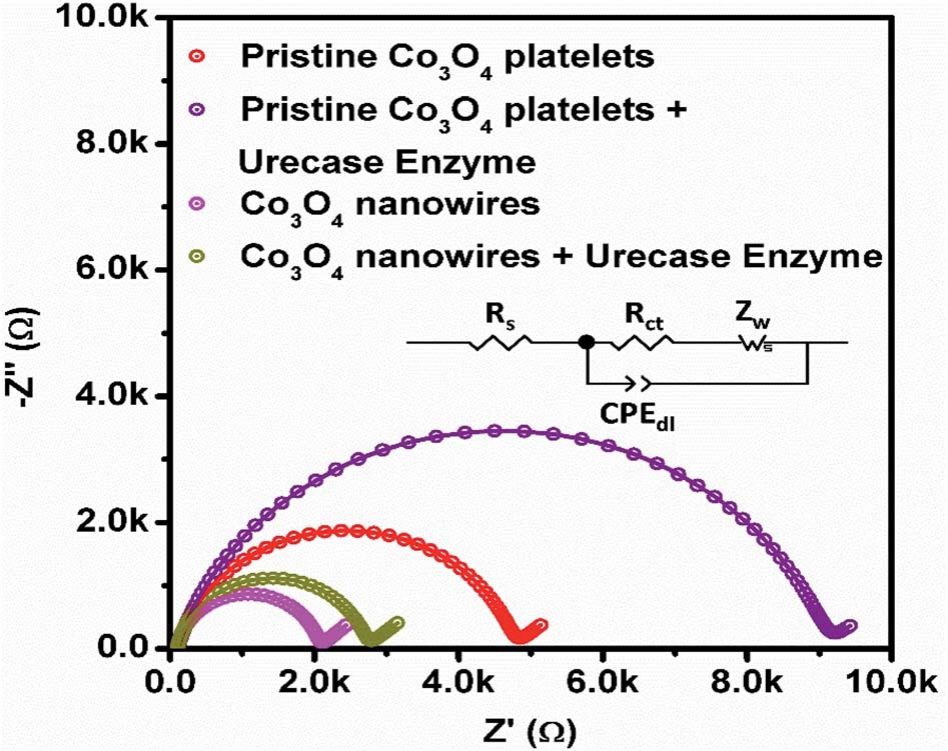
EIS Nyquist plots of pristine Co_3_O_4_ platelets and Co_3_O_4_ nanowires with and without enzyme in 0.1 mM uric acid in 0.01 M phosphate buffer solution at pH 7.4.

Fig. 10 shows typical Nyquist plots that demonstrate charge transfer during the potentiometric measurement of uric acid, and the inset shows the fitted equivalent circuit with well-defined elements. The Co_3_O_4_ nanowires show fast charge transfer, as observed from the small arc radius of the Nyquist plot, suggesting that the nanowires have better electrical communication channels that the platelet-like structure of Co_3_O_4_. However, after the immobilization of uricase, we see a slight increase in impedance due to the insulating features of uricase and glutaraldehyde, and these observations are in full agreement with previously published work.^66^ Furthermore, the estimated value of impedance for the Co_3_O_4_ nanowires is lower than the platelet-like structure of Co_3_O_4_, as given in Table 2, verifying the above claims based on the fast charge-transfer shown by the Co_3_O_4_ nanowires. The analytical performance of uricase-immobilizing nanowires obtained using cotton silk was also evaluated through the percentage recovery method, as given in Table 3. The obtained percentage recovery results were found to be suitable and they suggest that the proposed structure could be applied practically to uric acid determination. A comparison of the obtained results with recently published results for uric acid sensing is given in Table 4.^67–72^ The obtained results from the Co_3_O_4_ nanowires are superior in terms of the wide linear range, limit of detection, and fast response time, revealing the role of cotton silk in boosting the co-catalytic role of Co_3_O_4_ nanowires in the presence of uricase for the oxidation of uric acid.

**Table 2:** EIS fitted charge-transfer resistance values for Co_3_O_4_ nanostructures

Material	Charge-transfer resistance (ohms)
Pristine Co_3_O_4_	4654
Pristine Co_3_O_4_ + uricase	8992
Co_3_O_4_ nanowires	1950
Co_3_O_4_ nanowires + uricase	2606

**Table 3:** Percentage recovery of uric acid using uricase-immobilizing Co_3_O_4_ nanowires

Experiment no.	Added (mM)	Found (mM)	% recovery
1	3	3.1	103.03
2	4	4.05	101.25
3	5	4.98	99.6

**Table 4:** A comparison of recently reported potentiometric uric acid biosensors with the presented results

Sensing material	Linear range (mM)	Limit of detection (mM)	Response time (s)	Reference
RuO_2_	0.1–0.5	N/A	N/A	67
MBs–uricase/rGO/NiO	0.1–0.5	0.057	N/A	68
AgNW–uricase/rGO/NiO	0.1–0.5	0.068	N/A	69
ZnO nanowires	0.001–1.0	N/A	6–9	70
ZnO nanotubes	0.005–1.5	N/A	8	71
ZnO nanoflakes	0.0005–1.5	N/A	8	72
**Co**_**3**_ **O**_**4**_ **nanowires**	**0.0005–10**	**0.0001**	**1**	**Present work**

## 4. Conclusions

The abundant hydroxyl groups of cotton silk have been utilized for the fast nucleation/growth of well-oriented Co_3_O_4_ nanowires *via* a hydrothermal method. The Co_3_O_4_ nanowires, as confirmed *via* XRD and SEM images, were composed of only cobalt and oxygen as the main elements. The prepared Co_3_O_4_ nanowires exhibited a large surface area, which favored the high loading of the enzyme uricase through physical adsorption. The strong binding of uricase with the Co_3_O_4_ nanowires enabled the fabrication of a sensitive and selective potentiometric uric acid biosensor that could operate in 0.01 M phosphate buffer solution at pH 7.4. A wide linear working range from 5 nM to 10 mM (UA) and a low limit of detection of 1.0 ± 0.2 nM were reported for the presented uric acid biosensor. Important analytical parameters, such as reproducibility, stability, response time, and selectivity, were found to be satisfactory. The recovery method was used to verify the practical application of the fabricated biosensor, and it was found to perform satisfactorily. The obtained performance suggests that this uric acid biosensor based on uricase-immobilizing Co_3_O_4_ nanowires could be used as a potential sensor for the determination of uric acid. The use of cotton silk as a source of abundant hydroxyl groups for the fast nucleation of metal-oxide nanostructures could be of great interest for scientists and researchers for various applications.

## Conflicts of interest

The authors declare that there are no conflicts of interest related to this research work.

## Supplementary Material
